# Complications and risk management in the use of the reaming-irrigator-aspirator (RIA) system: RIA is a safe and reliable method in harvesting autologous bone graft

**DOI:** 10.1371/journal.pone.0196051

**Published:** 2018-04-26

**Authors:** Patrick Haubruck, Julian Ober, Raban Heller, Matthias Miska, Gerhard Schmidmaier, Michael C. Tanner

**Affiliations:** HTRG–Heidelberg Trauma Research Group, Center for Orthopedics, Trauma Surgery and Spinal Cord Injury, Trauma and Reconstructive Surgery, Heidelberg University Hospital, Heidelberg, Germany; UNITED KINGDOM

## Abstract

**Background:**

Autologous bone grafting (ABG) remains the gold standard for augmentation of bone defects. The RIA system has become more prevalent, but evidence regarding risk management and complications remain scarce. This study presents the risk management and complications associated with RIA in the largest single-center case series to date.

**Methods:**

All records, operative notes, lab data and radiographs of patients receiving a RIA procedure at Heidelberg´s University Hospital between 01/01/2010 and 31/12/2016 were reviewed. Multivariate logistic regression models adjusting for clinically relevant covariates were used to examine the respective relevance regarding the presence and absence of prolonged postoperative pain (PPP).

**Results:**

A total of 341 RIA procedures on 306 patients were performed at our level-1 trauma center. The femur was the main donor site (98.53%; N = 336) whereas only in 1.47% (N = 5) the tibia was utilized. A total of 11 patients showed a relevant loss of hemoglobin requiring blood transfusion. A total of 22 patients suffered from PPP directly associated with the RIA procedure resulting in prevalence of 6.45%. The 6 major complications in our study were of diverse origin and all intraoperative complications took place in the early phase of the RIA procedure in our center (2010–2013). Our data revealed influence of sex (p = 0.0459) and age (p = 0.0596) on the criterion PPP. The favored model including sex and age resulted in an AUC of 66.2% (CI: 55.5%-76.9%).

**Conclusion:**

Perioperative blood loss remains a prevalent complication during RIA reaming. In addition, PPP occurs with a prevalence of 6.45%. This study showed a complication rate of 1.76%, emphasizing RIA´s overall safety and furthermore highlighting the need for vigilance in its application and prior extensive hands-on training of surgeons. Level of Evidence: II.

## Introduction

Bone defects remain a challenging problem in orthopedic surgery [1]. They are common and caused by various pathologies like failed fracture healing, tumors and metastases, complex multi-fragmentary fractures and debridement after osteomyelitis. Autologous bone graft (ABG) remains the gold standard for augmentation of these defects [2]. The common donor site remains the iliac crest. However, complications such as donor-site morbidity, pain and quantitative limitations are well-documented [3–6]. Alternatives such as allogenic bone, demineralized bone matrix and bone substitutes are generally inferior regarding their osteogenic, osteoinductive and osteoproductive properties when compared to ABG [7, 8]. Hence the need for a reliable alternative with fewer complications arose.

Long-bone fractures requiring stabilization heal faster and more reliably when nail-insertion is preceded by reaming of the intramedullary canal [9]. However, reaming is associated with fat embolism (that can lead to respiratory distress [10]), as well as disturbances in intraosseus blood supply through excessive heat production [11, 12]. To reduce these adverse effects, a negative pressure reaming device was developed and validated by K.M. Stürmer in the 1980s [13]. To further reduce adverse effects in reaming, the cutting head was then cooled by fluid, which also transports the reaming debris from the intramedullary canal [3]. Thus the device was named reamer/irrigator/aspirator (RIA) [14]. Acquisition of reaming material was coincidental and interestingly studies investigating this debris found viable cells capable of initiating new bone formation [3, 15–17]. Hence RIA debris became a source of ABG. Multiple studies analyzed this material and demonstrated high concentrations of important growth factors (FGFa, PDGF, IGF-I, TGF-b1 and BMP-2) [3, 9, 18] therein, comparable to that from the iliac crest [3].

Due to its multiple advantages, the RIA system has become more common [4] and its use has been further expanded to the management of long bone infections [19]. Despite being established as a relatively safe method [20–22], evidence regarding the risk management and complications remains scarce in large patient cohorts. In this study we present the risk management and complications associated with the clinical use of RIA in the largest single-center case series to date.

## Material and methods

All medical records, operative notes, lab data and radiological imaging of patients receiving a RIA procedure at our hospital between 01/01/2010 and 31/12/2016 were reviewed regarding intra-operative complications, post-operative infection, pain, excessive bleeding, fracture and patient satisfaction. During the initial assessment patient data was not anonymized. However, not anonymized data was solemnly assessed by the treating and certified surgeons from our department and immediately after initial assessment patient data was fully anonymized prior to further analysis. Therefore, the ethics committee waived the requirement for informed consent and approval was received from the ethics committee of the University of Heidelberg (S-262/2017). This study was performed in concordance with the Declaration of Helsinki. Patient demographics were stratified to age, sex, donor site and number of performed RIA procedures. Complications were classified into major (infection, subsequent fracture and broken surgical instruments; CMP) and minor complications (bleeding requiring blood transfusion (BLD) and PPP).

All statistical calculations were performed with R version 3.2.3 [23] using the packages”ggplot2” [24] to create figures and “pROC” [25] for receiver operator characteristics (ROC) analysis corresponding to our previous studies [26, 27]. The Chi-square test was used to assess statistically significant differences in sex, BLD, CMP and PPP. For differences of the variable age regarding the criterion presence and absence of PPP, the parametric students T-test for independent samples was used. Primary measure for the predictive performance of any logistic regression model was the area under the curve (AUC) of the ROC-curve. The level of significance (α) was set at 5%.

## Results

### Patient characteristics

**Fig 1:**
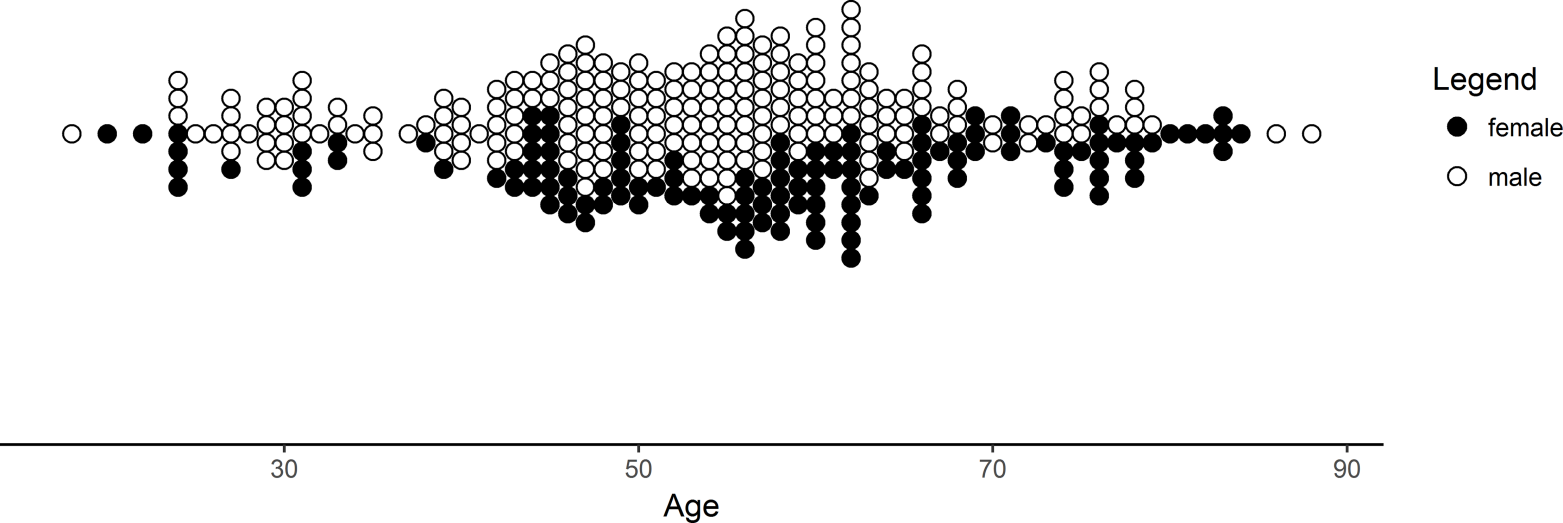
Number of RIA procedures. Number of RIA procedures is presented after stratification by sex and age.

Between 01/01/2010 and 31/12/2016 341 RIA procedures on 306 patients were performed at our level-1 trauma center. Indication for all RIA procedures was ABG of an osseous defect, performed in 63.1% on male patients (215 procedures on 193 men) and in 36.9% in female patients (126 procedures on 113 women), who were an average of 54 ± 14.12 years old (Table 1). Further stratification revealed that patients aged 50–59 years received most of the RIA procedures. Additional data concerning the distribution of RIA procedures in context with age and sex of patients can be found in Fig 1.

**Table 1:** Characteristics of procedures.

Sex	N RIA (pat; %)	Pat. RIAx1 (%)	Pat. RIAx2 (%)	Pat. RIAx3 (%)	Pat. RIAx4 (%)	Donor Site Femur	Donor Site Tibia		Tibia antegrade	Tibia retrograde	Average Age (SD)
Male	215 (193; 63.1%)	173 (56.5%)	19 (6.2%)	-	1 (0.33%)	211	4		1	3	52 ± 14.11
Female	126 (113; 36.9%)	100 (32.7%)	13 (4.2%)	-	-	125	1			1	57 ± 13.81
Total	341 (306)	273 (89.2%)	32 (10.4%)	-	1 (0.33%)	336 (98.53%)	5 (1.47%)		1	4	54 ± 14.12

Data is presented as total number of RIA procedures and in parenthesis the number of patients as well as percentage of the total study collective. N RIA = Number of RIA procedures; Pat. = Patient; Age in years is presented as mean ± standard deviation.

### Surgical approach and use of RIA

Preoperative radiological imaging of the selected RIA site was always performed to plan the procedure and evaluate bone quality at the donor site. All RIA procedures were performed by fully trained faculty members. The femur was the main RIA donor site (98.53%; N = 336) whereas only in 1.47% (N = 5) the tibia was utilized (Table 1). Surgical approach to the femur was in all cases antegrade; here, the standard piriformis entry [28] was carried out. In the tibia one case was via infrapatellar approach [29], all others were retrograde during ankle arthrodesis (Table 1). Positioning guide wires was diligently monitored by fluoroscopy to guarantee central reaming. All patients received single-shot antibiotics as per standard operative protocol. Administered antibiotic regarding prevention of surgical site infection was 1.5 grams of cefuroxime intravenously. 33 patients received repeated RIA procedures after an average of 13 ± 11.2 months and in all cases the femur was reamed (Table 2). 32 patients underwent the RIA procedure twice and one patient four times (Table 1). Therefore, a total of 35 repeated RIA procedures were conducted.

**Table 2:** Characteristics of repeated RIA procedures.

			Donor site in revision surgery	
Sex	N mult. RIA	Time to repeated RIA (months)	Ipsilateral	Contralateral
Male	20	10.9 ± 9.7	9	13
Female	13	16.4 ± 12.9	5	8
Total	33	13 ± 11.2	14	21

32 patients received a second RIA procedure and 1 patient received three repeated RIA procedures. Hence, 35 donor sites in revision surgery were utilized. N Pat. mult. RIA = Number of patients that received multiple RIA procedures; Pat. = Patient; Time to repeated RIA in months is presented as mean ± standard deviation.

### Complications

Complications were stratified into two main categories (Table 3).

1. According to Younger and Chapman [30] major complications were classified as either requiring additional operations, prolonging hospitalisation or causing significant permanent sequelae/disabilities [31].
2. Minor complications were PPP and BLD.

**Table 3:** Summary of complications.

Sex	Major complications (%) (CMP)			Minor complication Hb-loss (%) (BLD)			Minor complication postoperative pain (%) (PPP)		
	Total	Donor Site Femur	Donor Site Tibia	Total	Donor Site Femur	Donor Site Tibia	Total	Donor Site Femur	Donor Site Tibia
Male	3 (1.4%)	3	0	7 (3.25%)	7	0	10 (4.65%)	9	1
Female	3 (2.38%)	3	0	4 (3.17%)	4	0	12 (9.52%)	12	0
Total	6 (1.76%)	6	0	11 (3.23%)	11	0	22 (6.45%)	21	1

Data is presented as absolute number of complications and percentage associated with the total RIA procedures. Hb = hemoglobin

### Minor complications

**Fig 2:**
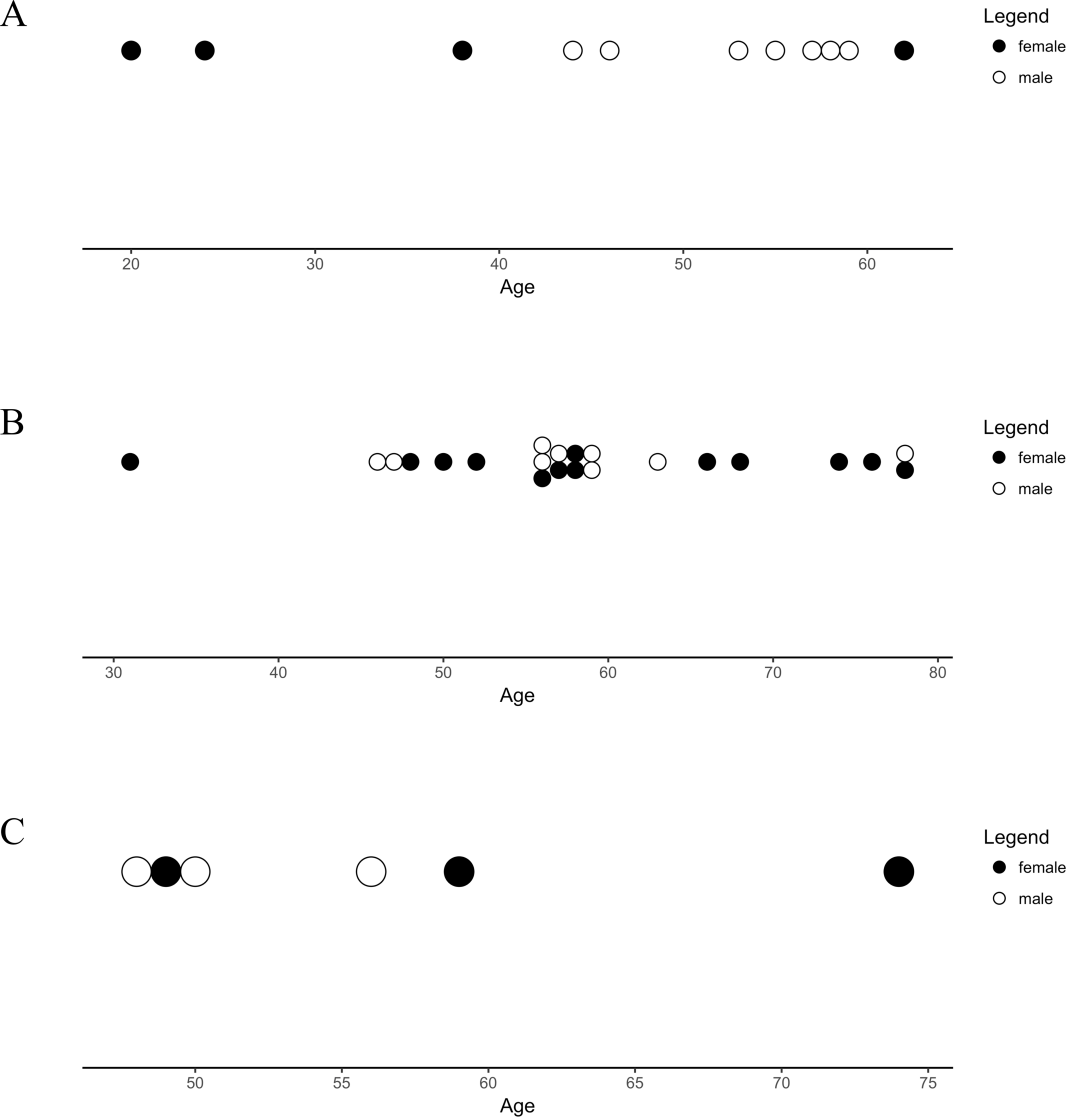
Number of all complications. (A) Number of patients that suffered from relevant peri-operative blood loss stratified by age and sex. (B) Number of patients that suffered from prolonged post-operative pain stratified by age and sex. (C) Number of major complications subsequent to stratification by age and sex.

A total of 11 patients required transfusions. Indications were based on hemoglobin-levels, cardiovascular risk-factors and clinical signs of anemia as per current guidelines [32]. Interestingly, most patients suffering from blood loss were aged 50 to 59. Nonetheless, there was no significant statistical influence of age or sex regarding the risk for blood loss (Table 4 and Fig 2A).

**Table 4:** Characteristics of patients suffering from BLD and PPP.

Case	Sex	Age (Year)	Donor Site	Approach	Hb-loss	Duration of pain (months)
1	m	59	Femur	Antegrade	6.3 g/dl	
2	m	53	Femur	Antegrade	6.6 g/dl	
3	m	46	Femur	Antegrade	9.2 g/dl	
4	m	58	Femur	Antegrade	5.6 g/dl	
5	m	54	Femur	Antegrade	2.8 g/dl	
6	m	44	Femur	Antegrade	7.5 g/dl	
7	m	57	Femur	Antegrade	8.9 g/dl	
8	f	62	Femur	Antegrade	4.9 g/dl	
9	f	24	Femur	Antegrade	5.8 g/dl	
10	f	20	Femur	Antegrade	7.1 g/dl	
11	f	38	Femur	Antegrade	3.5 g/dl	
1	m	57	Femur	Antegrade		12
2	m	63	Femur	Antegrade		2
3	m	48	Femur	Antegrade		1
4	m	56	Femur	Antegrade		1
5	m	46	Femur	Antegrade		24
6	m	59	Femur	Antegrade		1.5
7	m	56	Tibia	Antegrade		5
8	m	59	Femur	Antegrade		3
9	m	78	Femur	Antegrade		12
10	m	47	Femur	Antegrade		8
11	f	66	Femur	Antegrade		12
12	f	56	Femur	Antegrade		7
13	f	58	Femur	Antegrade		12
14	f	31	Femur	Antegrade		7
15	f	58	Femur	Antegrade		48
16	f	50	Femur	Antegrade		1
17	f	74	Femur	Antegrade		9
18	f	76	Femur	Antegrade		3
19	f	78	Femur	Antegrade		1.5
20	f	57	Femur	Antegrade		1
21	f	52	Femur	Antegrade		3
22	f	68	Femur	Antegrade		48

In our follow-up all patients were evaluated regarding post-operative pain and duration of post-operative pain was noted. Furthermore, Hb-loss is defined as drop in hemoglobin pra-operative to post-operative. Hb = hemoglobin; m = male; f = female

PPP at the donor-site was defined as procedure-related pain persisting 4 weeks or longer, discriminating between RIA and other procedures. Therefore, PPP was assessed initially 6 weeks subsequent to the latest RIA procedure and hereafter PPP was monitored at each follow-up visit. 22 patients suffered from PPP directly associated with the RIA procedure. (Table 4 and Fig 2B)

#### Binary logistic model

**Fig 3:**
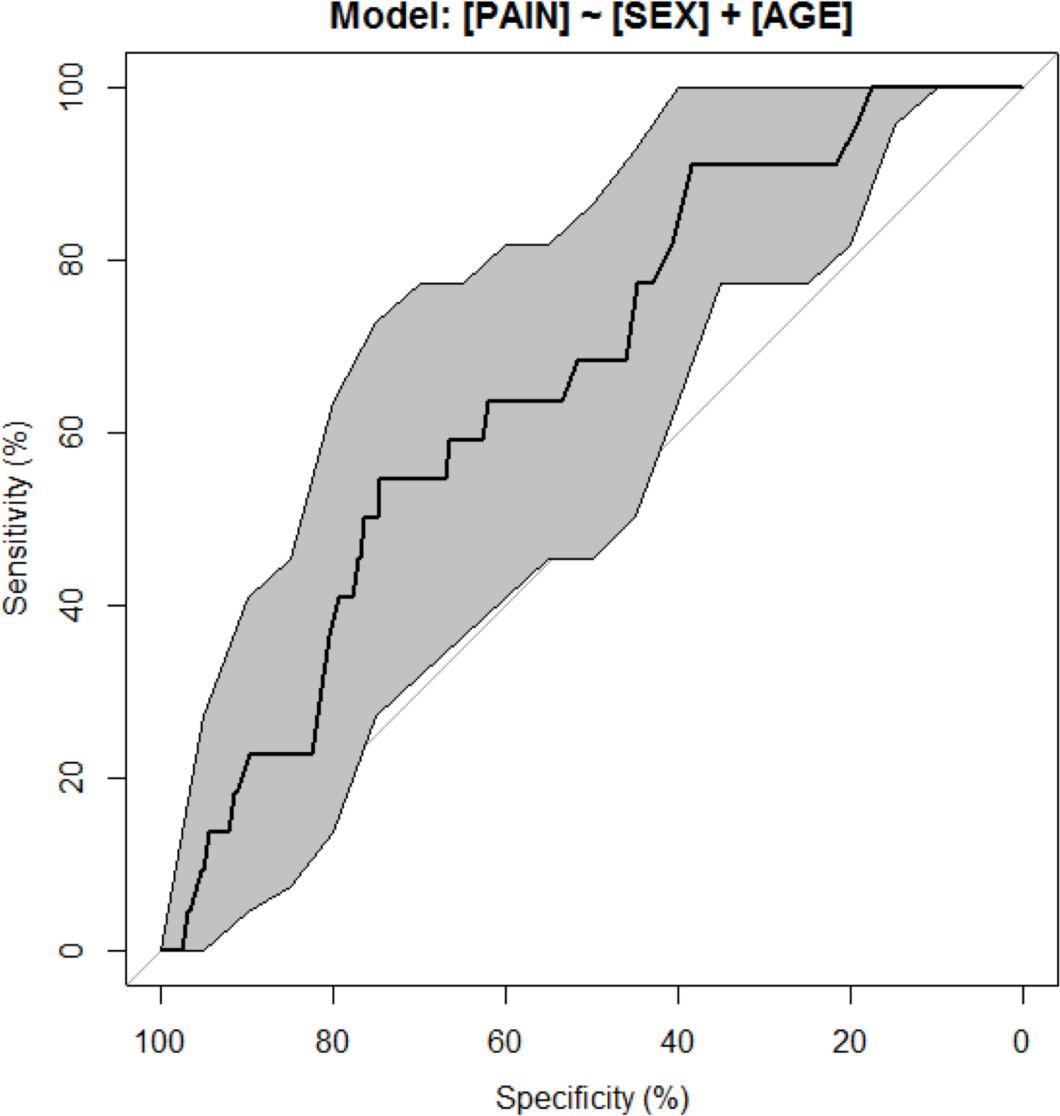
ROC. ROCs of the binary regression model. CI for marked as grey area in both directions.

Considering differences within the collective regarding the criterion PPP, a binary logistic regression model was used corresponding to our previous studies [26, 27]. CMP and BLD were excluded due to missing subset cases and thus limited additional information. A ROC analysis was performed based on the scores of the respective model. Age and sex were included in the final model. Our data revealed influence of sex (p = 0.0459 (ChiSquare test) and age (p = 0.0596 (t-test)) on the criterion PPP. The ROC curve for the resulted logistic regression model is shown in Fig 3. The regression model including has an area under the curve (AUC) of 0.66. The corresponding point estimates, 95% confidence intervals and p-values for the adjusted odds ratios are given in Table 5, based on the univariate regression analysis.

**Table 5:** Model characteristics.

			Confidence Interval	
Variables	Adj. Odds ratio	p-Values	0.025	0.975
SEX	0.419	0.057	0.166	1.02
AGE	1339	0.206	0.861	2.138

The odds ratios of variables included the preferred logistic regression model.

### Major complications

The 6 major complications were of diverse origin. 4 cases arose from technical difficulties with the RIA system. 3 patients suffered unintentional erosions of the femoral cortex both distal-laterally (1 of 3) and proximal-medially (2 of 3), despite diligent checks via c-arm in numerous planes. They were treated with a femoral nail, a PFNA and a LISS Femur plate, respectively. 1 patient suffered a dislocation of the drill from the RIA-system with debris from its tabs persisting in the proximal femur. One patient sustained substantial intraoperative bloodloss, necessitating transfusion of 13 units of blood and 12 FFPs. Although no local injury could be identified intraoperatively, she later developed a large haematoma in the proximal femoral area that needed to be evacuated. Furthermore this patient developed a deep infection of the soft tissue and necrosis of the femoral head, later treated with a total hip replacement. The last patient had an unknown patent foramen ovale (PFO), developing an embolism of his left A. cerebri media. Here all initial defects resolved over time, allowing him to return to his workplace, leaving him with slight cognitive impairment. It implicates that despite its primary developmental intentions, RIA may not always prevent this complication of reaming.

Interestingly, all complications secondary to intraoperative difficulties took place at an early stage of introduction of the RIA procedure (2010–2013) (Table 6, Fig 2C). We feel this emphasizes the need for extensive prior training and vigilant application of RIA to prevent unnecessary harm to patients.

**Table 6:** Characteristics of patients suffering from CMP.

Case	Sex	Age (Year)	Donor Site	Approach	Complications	Postoperative day	BMI	Year OP
1	m	56	Femur	Antegrade	Metaphyseal femur fracture	8	27.8	2010
2	m	48	Femur	Antegrade	Break off of RIA	intraoperative	29.36	2013
3	m	50	Femur	Antegrade	Fat embolism of left site A. cerebri media	10h postoperative	44.77	2012
4	w	49	Femur	Antegrade	Intraoperative weakening of dorsomedial femur corticalis	intraoperative	34.39	2010
5	w	74	Femur	Antegrade	postoperative soft tissue infection+ necrosis of femoral head	1 month postop.		2010
6	w	59	Femur	Antegrade	subtrochanteric femur fracture	2	27.34	2011

In one patient retrospective evaluation of the BMI was not possible. m = male; f = female; BMI = body mass index; CMP = major complications; RIA = Reaming Irrigator Aspirator

## Discussion

Osseous defects remain a challenge for surgeons. Despite research efforts towards an equivalent alternative, ABG remains the gold standard for augmenting these defects [2]. Reasons are its superiority regarding osteoconductive, osteoinductive and osteoproductive properties compared to allogenic bone, demineralized bone matrix and bone substitutes [7, 8]. However, ABG is limited in source and shows morbidity at the harvest site [3]. Consequently, over the years the RIA system proved a reliable alternative by harvesting ABG from the medullary canal of long bones. More importantly RIA has shown decreased morbidity at the harvest site and none of the complications at the iliac crest [13–17]. Still evidence regarding complications and risk management remains scarce. We sought to determine these factors associated with the use of RIA. To our knowledge this is the largest single center case study of RIA graft cases in literature [4].

Despite being developed as a procedure to reduce the prevalence of fat emboli secondary to intramedullary reaming and showing promise as a safe method for harvesting ABG, studies have reported various complications occurring when utilizing RIA. Treatment of large size osseous defects shows a relevant loss of blood. Harvesting from the iliac crest increases average intraoperative blood loss by 474 ml [31]. Data regarding blood loss associated with RIA is scarce. Extensive review revealed merely two relevant studies. McCall et al. reported an average hemoglobin decrease of 4,3 g/dL in patients undergoing RIA [33], Han et al. an average of 3,15 g/dL in a collective of 54 patients with RIA [20]. Our study shows blood loss is a relevant complication during the intraoperative use of RIA. Blood loss requiring transfusion occurred in 3.23% of RIA procedures (all from the femur). Average decrease of hemoglobin in patients requiring transfusion was 6.2 g/dL, confirming results previously gathered in smaller collectives.

Regardless the number of patients suffering from relevant blood loss, data from this current study gave us reason to believe that patients at a high-risk for anemia-associated co-morbidities [34] may benefit from intraoperative auto-transfusion of the blood lost due to the RIA system.

PPP is highly prevalent when grafting from the iliac crest and studies show decreased acute, intermediate and chronic PPP in RIA compared to the iliac crest [35–37]. However, a small number of patients suffering from chronic donor site pain is well documented for both RIA and iliac crest bone graft [20]. Interestingly there is no evidence regarding the prevalence of PPP subsequent to RIA [31].

22 patients (21 femur and 1 tibia) suffered from long-term donor-site pain, resulting in prevalence of 6.45% with PPP after RIA. To our knowledge this is the first study providing reliable data regarding prevalence of PPP after RIA in long bones. Further, our data indicated an influence of sex on the occurrence of PPP. Despite prevalence of PPP subsequent to RIA being small the intraoperative administration of local anesthetics should be considered to prevent chronification of pain; furthermore, PPP management should provide analgesia according to WHO recommendations.

RIA was developed to decrease intramedullary pressure and the risk of fat embolism [38]. Multiple experimental studies (animal and cadaveric) confirm RIA`s success in decreasing intramedullary pressure of reamed long-bones [39–43]. A protective effect regarding fat embolism has been established by quantifying embolic load and systemic response [41, 43]. However, to date, no clinical data supports these experimental findings.

One patient suffered an embolic occlusion of the medial cerebral artery due to embolism associated with RIA and PFO. Despite being an extremely rare complication, due to its severe impact on patients caregivers should be aware that fat embolism subsequent to RIA may still occur in patients with appropriate risks. In particular, patients with PFO should be monitored closely and preoperative occlusion considered.

Due to its versatility, the use of RIA has been expanded from harvesting ABG to treating intramedullary osteomyelitis [19] and studies have supported efficacy of this treatment [44, 45]. However, so far no data exists regarding the risk of infections subsequent to RIA reaming. In our study one patient suffered from a deep soft tissue infection secondary to RIA. Despite being a rare condition, intraoperative administration of antibiotics and close monitoring of wounds should be performed regularly to further decrease the prevalence of this complication.

RIA technique has been well described in several studies [46, 47] and a steep learning curve is associated with its use [19]. Technical errors remain a potential source for complications, the most serious potential complication being iatrogenic femur fracture [46]. To prevent this, meticulous intraoperative technique and correct patient selection have proven necessary [38]. RIA reaming should be avoided in patients with significant osteoporosis/osteopenia unless post-operative stabilization is planned [19]. Appropriate selection of the RIA head is mandatory. According to Giannoudis et al. a reamer head no more than 2mm wider than the smallest diameter of the isthmus should be used to prevent cortical thinning [46]. Accurate identification of the entry point should be confirmed by intra-operative fluoroscopy [38] and furthermore the correct placement of the guide wire is critical to avoid perforation of the medial proximal or anterior distal cortex [46]. Frequent intra-operative fluoroscopy is mandatory to prevent misguiding the guide wire, verify the position during reaming and evaluate the width of the cortexes [46]. Sufficient expertise is necessary to minimize complications [47].

We encountered three cases of technical errors predominantly in the early phases of RIA reaming in our hospital. The proximal dorsomedial cortex of the femur was weakened intra-operatively resulting in two post-operative fractures of the proximal femur as well as the ventrolateral cortex suffering from a fissure resulting in instability and pain. All complications were treated with surgical stabilization. This emphasizes the need for vigilance during RIA and prior extensive hands-on training of surgeons. To further substantially shorten the learning curve for the RIA device, special training should be implemented for each surgeon, preferably at a ‘center of excellence’ [46]. Previously reported overall complication rates associated with RIA reaming vary from 1.96% up to 6.2% [4, 35, 48]. To our best knowledge this study is the largest single center case study, showing an overall complication rate of 1.76%. Our data emphasizes the overall safety of this technique and implies a lower complication rate in a larger collective due to a crucial learning curve.

### Limitations

To our best knowledge this is the largest single center case study regarding clinical use of the RIA device. Nevertheless, our study has limitations.

Surgical treatment of non-unions is associated with high blood loss. Blood loss is multifactorial and despite best intentions to accurately correlate it with the RIA procedure, possible confounders are blood losses at the main surgical site and possible dilution due to variations in fluid management. All operative notes have been extensively studied. According to our institutional standard elevated blood loss at the main surgical site has to be reported in the operative note. However, after reviewing every operative note there was no evidence regarding any elevated blood loss at the main surgical site; whereas, additional objective methods to monitor local blood loss were not conducted. Thus, findings regarding blood loss may be confounded due to misinterpretations of the treating surgeon. However, due the standardized approach of reporting operative notes we believe this misinterpretation to be minimal and that this does not implicate the importance of our findings.

Pain is known to be highly subjective and reasons for PPP can be attributed to various pathologies. In this study postoperative pain was assessed by clinical evaluation of the patient and their subjective history. Association of postoperative pain with RIA reaming is challenging, nonetheless effective pain relief is crucial regardless of its derivation. This study was able to identify patients at risk for postoperative pain, therefore we believe that these findings add important knowledge to the literature in the field.

### Conclusion

Large-size osseous defects remain a challenge for surgeons and ABG from the iliac crest remains the gold standard for augmentation of these defects. The RIA system was introduced as a reliable alternative, enabling surgeons to harvest ABG from other locations. Despite being established as relatively safe method, evidence regarding the risk management and complications remains scarce in large patient cohorts. To our knowledge this is the largest single center case study currently available. Our study’s data identifies relevant complications and offers possible approaches for their prevention and successful risk management.

Perioperative blood loss remains a common complication during RIA reaming. Data from the current study led us to believe that high-risk patients for anemia-associated comorbidities may benefit from intraoperative autotransfusion. Additionally, PPP occurs subsequent to harvesting the iliac crest as well as RIA, however in the latter to a lesser extent. This study provided a prevalence of 6.45% of postoperative pain subsequent to clinical use of RIA. To our knowledge this is the first study providing reliable data regarding prevalence of PPP subsequent to the RIA procedure in long bones. Despite prevalence of PPP subsequent to RIA being small the intraoperative administration of local anesthetics should be considered to prevent chronification of pain. While RIA´s developmental intention was reduction of fat embolism one patient suffered from a cerebral embolism most likely due to fat embolism through PFO. Despite being a rare complication, due to its severe impact on patients’ caregivers should be aware that emboli may still occur during RIA. Particularly patients with PFO should be closely monitored and preoperative occlusion should be highly considered.

Overall complication rate associated with RIA reaming varies from 1.96% up to 6.2% [4, 35, 48]. Our study showed a complication rate of 1.76% thereby emphasizing the overall safety of this technique and implies a lower complication rate in a larger collective due to a crucial learning curve. To further substantially shorten the learning curve for the RIA device special training at a center of excellence should be implemented for each surgeon.
